# Supplementary figures and images for: Diversity and habitat preferences of bdelloid rotifers in mosses and liverworts from beach forest along sand dunes in Thailand
Source: PeerJ. 2024 Dec 16;12:e18721. doi: 10.7717/peerj.18721 (PMC11657207; doi:10.7717/peerj.18721)

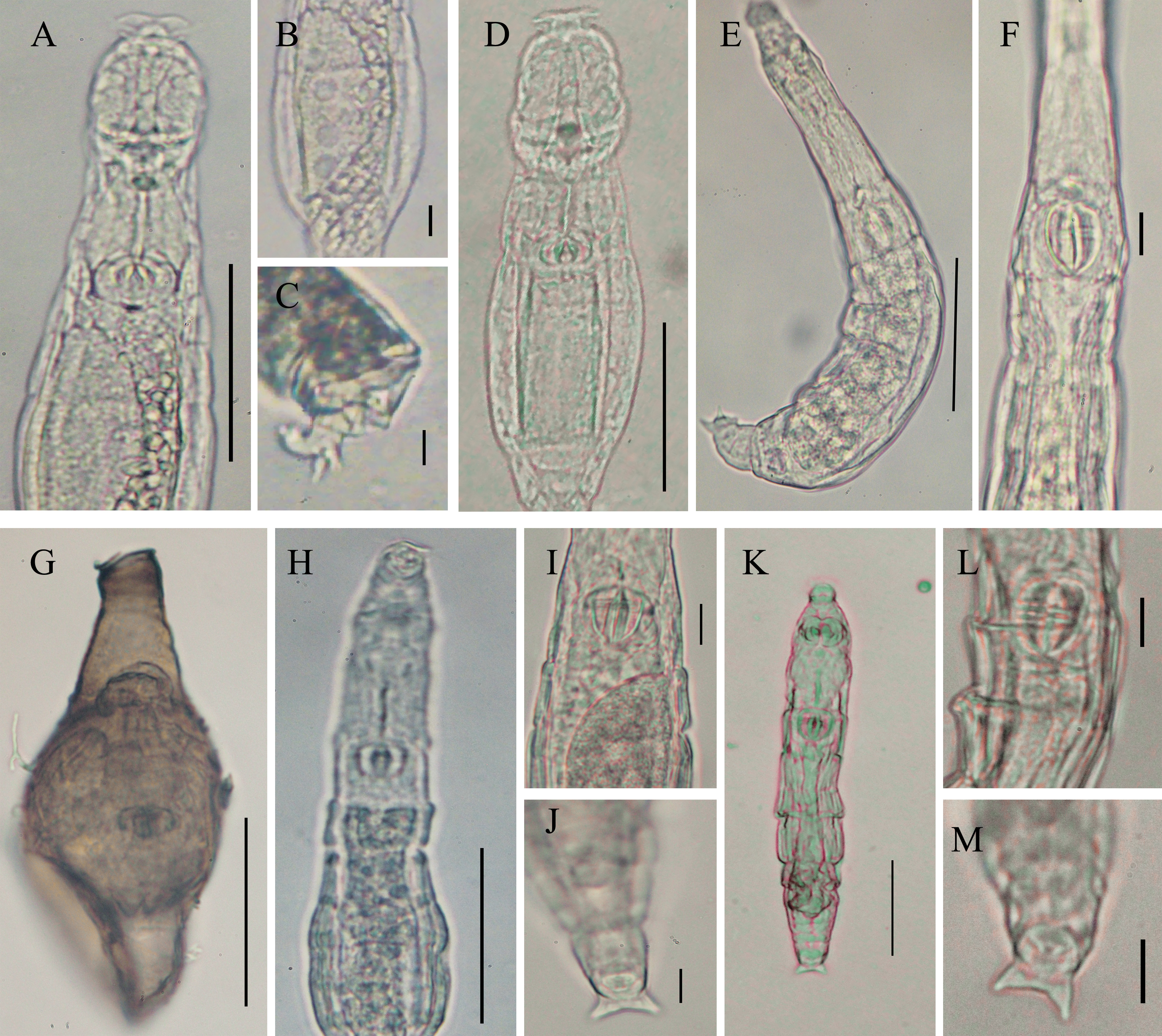

Supplement: Supplemental Information 2 — (A) creeping, dorsal view; (B) creeping trunk, dorsal view; (C) foot, spurs and toes, lateral view. Adineta vaga (D) creeping, dorsal view. Habrotrocha cf. alacris (E) creeping, dorsal view; (F) creeping trunk and trophi, dorsal view. Habrotrocha angusticollis (G) contracting, dorsal view. Habrotrocha bidens (H) creeping, dorsal view; (I) creeping trunk and trophi, dorsal view; (J) foot and spurs, dorsal view. Habrotrocha cf. brocklehursti (K) creeping, dorsal view; (L) creeping trunk and trophi, dorsal view; (M) foot and spurs, dorsal view. (scale bars: B-C, F, I-J, K-L = 10 µm; A, D-E, G-H, K = 50 µm). [file peerj-12-18721-s002.jpg]

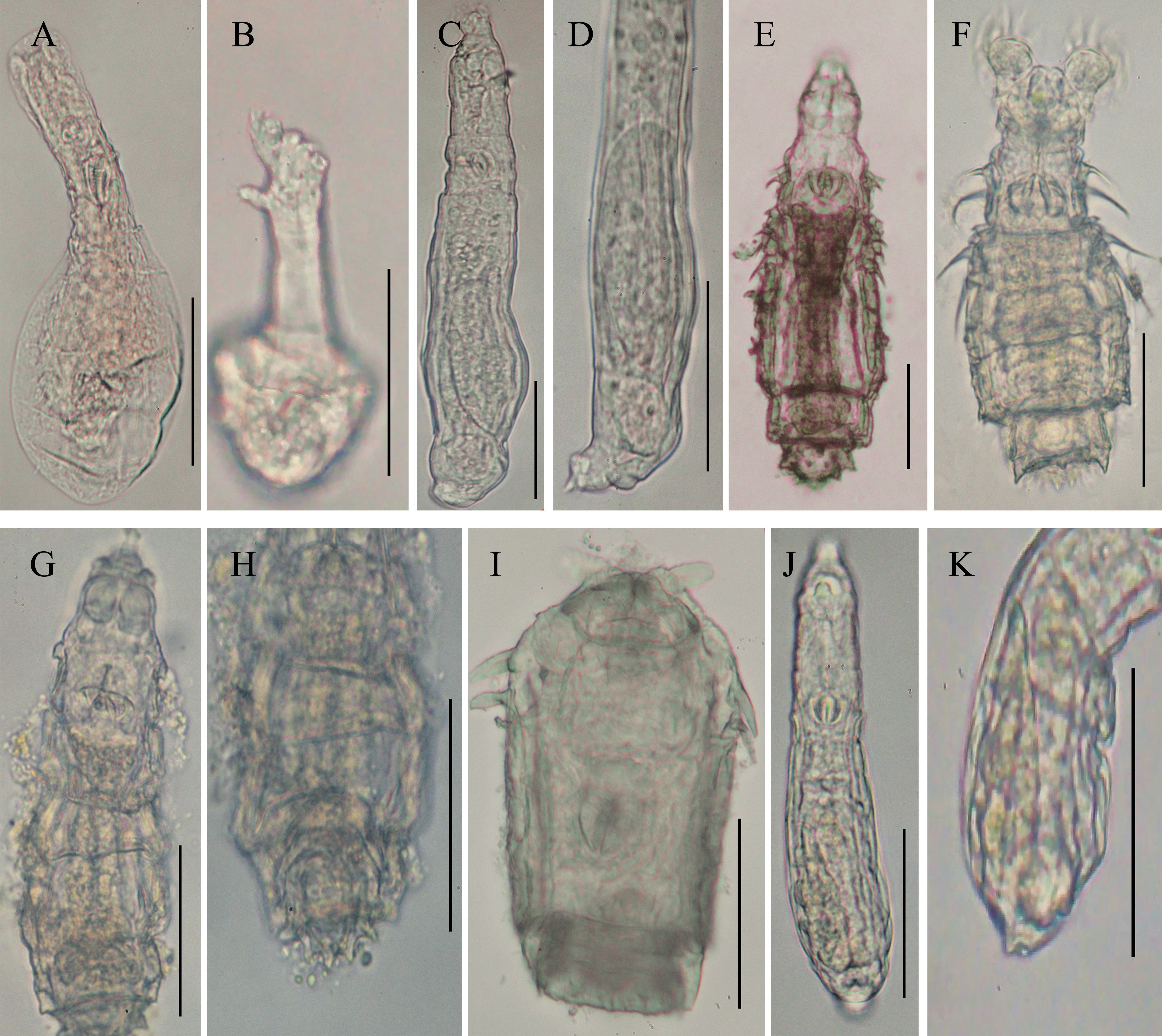

Supplement: Supplemental Information 3 — (A) creeping, lateral view; (B) feeding, lateral view. Habrotrocha gracilis (C) creeping, lateral view; (D) trunk and foot, lateral view. Macrotrachela multispinosa (E) creeping, dorsal view; (F) feeding, dorsal view. Macrotrachela papillosa (G) creeping, dorsal view; (H) trunk and foot, dorsal view. Macrotrachela pinnigera (I) contracting, dorsal view. Macrotrachela cf. plicata (J) creeping, dorsal view; (K) creeping trunk, lateral view. (scale bars: A-K = 50 µm). [file peerj-12-18721-s003.jpg]

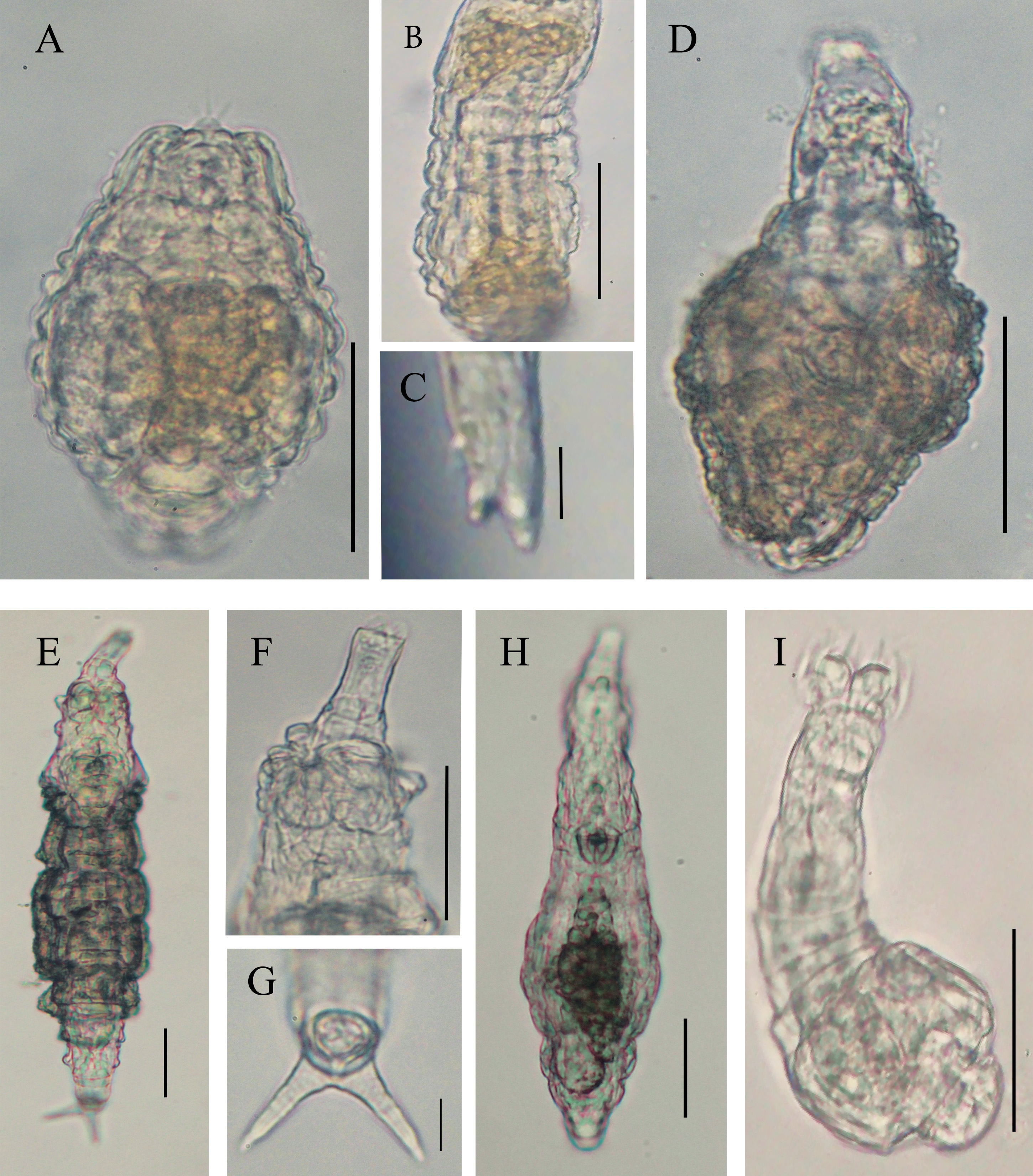

Supplement: Supplemental Information 4 — (A) contracting, dorsal view; (B) creeping trunk, dorsal view; (C) foot and spurs, lateral view; Philodina verrucosa (D) contracting, dorsal view. Rotaria sordida (E) creeping, dorsal view; (F) head, dorsal view; (G) foot, dorsal view. Scepanotrocha simplex (H) creeping, dorsal view; (I) feeding, dorsal view. (scale bars: C, G = 10 µm; A-B, D-F, H-I = 50 µm). [file peerj-12-18721-s004.jpg]
